# Editorial: Risk and preventive factors in necrotizing enterocolitis and its complications in premature infants

**DOI:** 10.3389/fped.2022.1056124

**Published:** 2022-10-21

**Authors:** María L. Couce

**Affiliations:** Department of Neonatology, University Clinical Hospital of Santiago de Compostela, RICORS, IDIS,,CIBERER, MetabERN, Santiago de Compostela, Spain

**Keywords:** amniotic fluid, enteral nutrition, intestinal perforation, prematurity, surgery

**Editorial on the Research Topic** Risk and preventive factors in necrotizing enterocolitis and its complications in premature infants By Couce ML. (2022) Front. Pediatr. 10: 1056124. doi: 10.3389/fped.2022.1056124

Necrotizing enterocolitis (NEC) is a potential complication of premature birth that leads to intestinal inflammation, bacterial invasion of the intestinal wall, formation of intramural gas bubbles (pneumatosis), and coagulative necrosis, and in advanced stages affects all intestinal layers. It is associated with high mortality rates (20%–30% of cases), which are higher at younger gestational ages and in infants who require surgical treatment, as well as high morbidity, including poor neurodevelopment (1, 2). The pathogenesis of NEC is not entirely understood (3): certain factors can act as triggers, while others can prevent it. Some are better described than others (4–7). Achieving a decreasing incidence of NEC could also help reduce the incidence of other inflammatory morbidities, including bronchopulmonary dysplasia and retinopathy of prematurity. These comorbidities remain mostly invariable in patients with NEC (8). Further research is required to develop novel therapeutic approaches that target the systemic inflammatory component of NEC and to devise new preventive strategies to reduce its incidence and prevalence in neonatal units.

This Special Issue of *Frontiers in Pediatrics*, “Risk and Preventive Factors in Necrotizing Enterocolitis and its Complications in Premature Infants” comprises 6 studies focusing on pathophysiological mechanisms, risk and preventive factors, surgical outcomes, and promising therapeutic developments. This collection of articles underscores the extensive interest in research on this topic.

Frid et al. (2021) compared the pathophysiological mechanisms in premature vs. full term infants with congenital heart disease (CHD), and found that both platelet counts and C-reactive protein (CRP) levels were higher in premature infants. Given that CRP and platelets are known acute phase reactants, this finding indicates a more robust inflammatory response in premature infants, and suggests that these patients may develop inflammation-based disease, whereas full-term CHD patients may be more likely to experience ischemic-related disease. In their study, Chen et al. (2022) investigated the molecular mechanisms underlying secondary intestinal stricture in NEC and sought to identify proteins or genes that could be targeted to effectively prevent or alleviate intestinal stricture. Interestingly, they observed that silent mating–type information regulation 2 homolog-1 (SIRT1) can regulate intestinal fibrosis and protect intestinal mucosal barrier function, and participates in the process of post-necrotizing enterocolitis stricture, suggesting a potential protective role of this protein in NEC.

The incidence of NEC could be reduced by expanding our knowledge of the corresponding protective, risk, and predictive factors. Alene et al. (2022) identified preeclampsia, premature rupture of the membranes, perinatal asphyxia, gestational age <32 weeks, and birth weight <1,000 g as independent predictors of NEC occurrence. A systematic review of the literature by Campos-Martinez et al. (2022) reports that the incidence of NEC can be reduced by supplementation of enteral nutrition with human milk oligosaccharides (with prebiotic and immunomodulatory effects); combined administration of probiotics (especially the combination of *Lactobacillus* spp. and *Bifidobacterium* spp.); supplementation of human milk with lactoferrin; and the use of donated milk fortified in accordance with the characteristics of the premature newborn. According to the same authors, a better understanding of new biomarkers, such as IL-6 and IL-17, would facilitate early identification of NEC and thereby help reduce its incidence.

Han et al. (2022) compared surgical outcomes between patients with perforated and non-perforated NEC, and found that infants with surgical NEC in the non-perforated group were more prone to bowel necrosis and a higher mortality rate than those in the perforated group. The authors conclude that Bell staging is not accurate for the diagnosis of severe NEC that requires surgical intervention.

In their contribution, Romee de Kroon et al. (2022) review experimental studies in animal models that have investigated the potential of amniotic fluid (AF)-based approaches for the prevention and/or treatment of NEC. Further research will be necessary to determine whether these proposed approaches will benefit neonates that have developed or are susceptible to NEC.

## Conclusion

This special issue provides a useful summary of progress made in the field of necrotizing enterocolitis, particularly in premature infants. The reported findings broaden our understanding of various aspects of this disease, better defining clinical phenotypes and expanding our knowledge of the pathophysiology of this disease. Nonetheless, clinical studies with longer follow-up periods will be necessary to establish recommendations that can improve the prognosis of this serious health problem in premature newborns.
